# 

**Published:** 1860-02

**Authors:** 


### ASSOCIATIONS—MEETINGS.

The Ohio Dental College Association will meet according to adjournment on Monday, February 20th, at 2 o'clock, P. M. It is a matter of regret that the meetings of this Association are not better attended by its members.

We have thought that there are two or three considerations that should induce all those interested to attend. There is pecuniary investment in which all the members are interested, and which should receive their attention.

And more than this is the object of the institution, viz., the training of those about to enter the profession ; this is a subject in which all the members of the profession are deeply interested, and one which they can control at pleasure, for *weal* or *woe.*

Professional education lies at the foundation, and should be fostered and sustained by all.

All who are interested in the profession should aid in sustaining and promoting its literature and education ; and yet it is discouraging to see how little interest is manifested by many. Every dentist should visit our Colleges and become familiar with, and take interest in their operations, and extend their cöoperation, and in this way increase their efficiency.

It is not just that the weight of these enterprises should rest wholly upon the shoulders of a few feeble instruments.

We hope that the members of the profession in the West, at least, will come in and let us have a full meeting of the Dental College Association.

The MississippiValley Association meets in the Ohio College of Dental Surgery, on Wednesday the 22d of February, at 10 o'clock, A. M., (not on the 21st, as stated in the minutes of the last meeting.)

Fellow practitioners, look back on the doings of this society for the past fifteen years, and see what it has done for the profession, and if in this, there is no inducement for you to attend its future meetings, we should expect to fail in presenting any other argument as an inducement to secure your attention.

We will meet the *veterans* of this society, and we hope to have many accessions to its ranks.

And we hope all who come will bring a *tribute.* We always regret to see any one come with any thing so tightly bound up it can't be opened out. T

☞ We have just received a "Dental Essay," containing sixteen pages, for popular reading, by Dr. J. F. Wilson, of Louisville, Ky.

This "little book" contains some valuable hints, but does not occupy the ground fully; but probably as well as sixteen small pages could do. We think this a good method of giving information to the people in regard to their teeth, and the best means of taking care of them.

Care should be taken that they do not partake of the character of advertisements or placards, than which nothing is more offensive, and more completely defeats the object that should be attained, in an enlightened community. T.

### THE COMMENCEMENT EXERCISES,

Of the present session of the Ohio College of Dental Surgery will be held in the first lecture room of the College on Wednesday evening, February 22, at 7½ P. M., when we hope to see the friends of the institution; and all who feel an interest in the progress of Dental science, are invited to be present.

### GOLD FOIL.

We have made some experiments with "Competition" Gold Foil from Sutton & Raynor, which is manufactured by them. It is quite adhesive, but like other foil requires annealing, and welds very perfectly, when worked with good points. Fillings made with it are susceptible of a very high finish, which is always an indication that its cohesion is good. The fillings we have made with this foil have not been excelled by any fillings we have made with any other preparation of gold. We would advise all who use the best foil they can get, to try this, and if it is uniform they will find a good article. T.

### REMOVAL.

We learn that Dr. H. A. McDaniel, a graduate of the Ohio College of Dental Surgery, for sometime a resident practitioner of Hopkinsville, Ky., and for the last few years, of Nashville, Tenn., has removed and permanently located at Huntsville, Alabama.

The citizens of Huntsville may congratulate themselves upon the acquisition of so thorough an operator; careful and skillful, and withal a high toned gentleman. T.

### THIRTEENTH VOLUME

OF THE

### DENTAL REGISTER OF THE WEST,

EDITED BY

**J. TAFT and GEO. WATT.**

**Issued Monthly. - - - $3 a year in Advance.**

**The Volume commences in July. CONTRIBUTIONS to the pages of the Register respectfully solicited.**

**Exchanges and Correspondents will please address "Dental Register," or J. TAFT, Cincinnati, O; Business communications to JNO. T. TOLAND.**

**The Register being issued Monthly is always fresh, and in advance of all others, therefore indispensable to the practitioner who desires to keep posted up with the new inventions and improvements which are daily developed.**

**Neither expense nor effort will be spared to give value and variety to its contents. Articles will be illustrated with well executed engravings, whenever they will add to the interest or assist in explanation.**

**Its extensive circulation and frequency of issue makes it the BEST DENTAL ADVERTISING MEDIUM in the United States.**

### TERMS OF ADVERTISING.

| | One Year. | 6 Mo. | 3 Mo. | 1 Insertion. |
|---|---|---|---|---|
| One Page, | $50 | $30 | $20 | $15 |
| Half. | 30 | 20 | 15 | 10 |
| Quarter. | 20 | 15 | 10 | 7 |
| One-Eighth. | 15 | 10 | 7 | 5 |

**JNO. T. TOLAND, Pub. and Proprietor,**

38 *West Fourth Street, Cincinnati, Ohio.*

